# Ocular surface symptoms and tear-film parameters in keratoconus: a systematic review and meta-analysis

**DOI:** 10.3389/fmed.2026.1797492

**Published:** 2026-10-15

**Authors:** Yang Cao, Yue Yang, Wei Deng, Nan Yang

**Affiliations:** 1 Department of Ophthalmology, The First Hospital of Hunan University of Chinese Medicine, Changsha, China; 2 First Clinical College of Traditional Chinese Medicine, Hunan University of Chinese Medicine, Changsha, China; 3 Department of Nephrology, The First Hospital of Hunan University of Chinese Medicine, Changsha, China

**Keywords:** dry eye disease, keratoconus, meta-analysis, ocular surface, OSDI, tear film

## Abstract

**Background:**

Ocular-surface abnormalities are increasingly recognized in keratoconus (KC), but the magnitude and consistency of these changes remain uncertain. We evaluated ocular-surface symptoms, tear-function parameters, and dry-eye-related categorical outcomes in patients with KC compared with non-KC controls.

**Methods:**

PubMed, Embase, Web of Science, and the Cochrane Library were searched from inception through September 1, 2026. Comparative studies reporting the Ocular Surface Disease Index (OSDI), fluorescein tear break-up time (TBUT), noninvasive TBUT (NITBUT), the Schirmer I test (SIT), or a defined dry eye disease (DED) classification were eligible. Meibomian gland dysfunction (MGD) and meibomian gland atrophy (MGA), when reported by otherwise eligible studies, were summarized as secondary ocular-surface findings. Two reviewers independently screened studies, extracted data, and assessed methodological quality using an adapted Newcastle–Ottawa Scale (NOS). Continuous outcomes were quantitatively pooled when the analysis unit and measurement methods were sufficiently defined and comparable; otherwise, study-level findings were summarized without calculating a pooled estimate. Random-effects models were estimated by restricted maximum likelihood with Hartung-Knapp adjustment.

**Results:**

Seven studies involving 359 participants with KC and 333 controls were included. OSDI scores were significantly higher in KC (MD, 19.45 points; 95% CI, 12.27 to 26.63; I^2^ = 79.9%). Fluorescein TBUT was shorter, although the pooled difference was not statistically significant (MD, −2.74 s; 95% CI, −7.81 to 2.32; I^2^ = 93.8%). All three studies reporting NITBUT found lower values in KC, but eye selection or data aggregation was unclear in two studies; therefore, a pooled NITBUT estimate was not calculated. SIT findings varied across studies, and a pooled estimate was not calculated because statistical independence of eye-level data and sufficiently comparable testing procedures could not be established across an adequate number of studies. The OSDI finding remained robust across sensitivity and leave-one-out analyses.

**Conclusions:**

Patients with KC had higher OSDI scores. The pooled difference in fluorescein TBUT was not statistically significant. Lower NITBUT and SIT values were reported in several studies, but pooled estimates for these outcomes were not calculated because of limitations in eye-level data handling and measurement comparability. Larger prospective multicenter studies using clearly defined analysis units and standardized tear-testing protocols are needed.

**Systematic Review Registration:**

https://www.crd.york.ac.uk/PROSPERO/, identifier CRD420261282391.

## 1 Introduction

Keratoconus (KC) is a progressive corneal ectatic disorder characterized by corneal thinning and steepening, irregular astigmatism, and progressive visual impairment (1). KC is increasingly recognized as a multifactorial disorder involving genetic, environmental, biomechanical, and cellular mechanisms rather than as an isolated structural abnormality (2). Alterations in stromal collagen organization and cross-linking, inflammatory signaling, and oxidative stress have all been implicated in disease development and progression (3). Environmental and behavioral factors may further interact with these processes; in particular, eye rubbing and atopy have been independently associated with KC (4).

Increasing attention has therefore been directed toward the ocular-surface environment in KC. Inflammatory mediators and immune alterations have been identified in the cornea and tear film of patients with corneal ectasia, while allergy and atopy may promote itching and repetitive eye rubbing (5). Consistent with this concept, higher tear concentrations of MMP-9 and HMGB1 and lower SOD3 levels have been reported in KC, supporting the presence of an altered inflammatory and oxidative ocular-surface microenvironment (6). *In vivo* confocal microscopy has also demonstrated reduced corneal nerve density, disorganization and increased tortuosity of the subbasal nerve plexus, and altered corneal sensitivity (7). Beyond epithelial and neurosensory changes, quantitative meibography has identified morphological abnormalities of the meibomian glands and associations between gland morphology, lipid-layer thickness, and KC severity (8). Collectively, these observations suggest that ocular-surface involvement in KC may extend across inflammatory, neurosensory, tear-film, and meibomian gland components.

Clinical evaluation of ocular-surface abnormalities in KC remains challenging because commonly reported outcomes assess different physiological domains and are not necessarily interchangeable. Fluorescein tear break-up time (TBUT) and noninvasive tear break-up time (NITBUT), for example, are obtained using different techniques and may yield systematically different values (9). A recent systematic review documented a broad spectrum of ocular-surface abnormalities in KC, including dry-eye-related changes, tear-film instability, and inflammatory alterations (10). Interpretation across studies is further complicated by differences in KC severity, contact-lens exposure, testing protocols, control-group selection, and analysis units, including participant-level, single-eye, and bilateral-eye data. These methodological differences may contribute to the inconsistency of reported ocular-surface findings and complicate quantitative comparison across studies.

Accordingly, this systematic review and meta-analysis aimed to evaluate differences in OSDI, fluorescein TBUT, NITBUT, and SIT between patients with KC and non-KC controls; to separately summarize categorical DED outcomes and additional secondary ocular-surface findings, including meibomian gland dysfunction (MGD) and meibomian gland atrophy (MGA); and to examine relevant methodological sources of heterogeneity.

## 2 Methods

### 2.1 Search strategy

PubMed, Embase, Web of Science, and the Cochrane Library were searched from inception through September 1, 2026. Search terms were adapted to the title/abstract or equivalent fields of each database. The search strategy combined terms related to keratoconus and dry eye disease, including ([Keratoconus OR Keratoglobus OR Pellucid Marginal Corneal Degeneration OR Pellucid Marginal Degeneration] AND [Dry Eye Syndromes OR Dry Eye OR Dry Eye Disease OR Evaporative Dry Eye OR Evaporative Dry Eye Disease OR Evaporative Dry Eye Syndrome]). No language restrictions were applied.

### 2.2 Eligibility criteria

Studies were eligible if they included patients with clinically, topographically, or tomographically diagnosed KC and a non-KC control group; reported at least one outcome of interest (OSDI, fluorescein TBUT, NITBUT, SIT, or a defined DED classification); and used a comparative observational design or provided separately extractable baseline data.

MGD and MGA were not used as eligibility outcomes; when reported by otherwise eligible studies, they were summarized as secondary ocular-surface findings.

Non-original reports, studies without an appropriate control group or extractable outcome data, and post-intervention studies without separable untreated or baseline data were excluded. Age and sex comparability, contact-lens exposure, allergy or atopy, KC severity, and previous ocular procedures were recorded as potential sources of heterogeneity.

### 2.3 Study selection and data extraction

Two reviewers independently screened records, assessed full-text articles, and extracted data using a standardized form; disagreements were resolved by consensus or a third reviewer.

Extracted data included study design, participant and eye counts, demographics, KC severity, contact-lens use, allergy or atopy, previous ocular procedures, control definition, study-eye selection, bilateral-eye handling, outcome definitions, measurement methods, and quantitative results. Missing information was recorded as not reported.

Mutually exclusive KC subgroups sharing a common control group were combined using established methods for continuous outcomes, with the control group counted once (11). For studies that appropriately accounted for inter-eye correlation, the reported adjusted estimate was used (12, 13).

### 2.4 Outcome-level eligibility for quantitative synthesis

OSDI, fluorescein TBUT, NITBUT, and SIT were evaluated separately; fluorescein TBUT and NITBUT were not combined because they represent methodologically distinct measurements. OSDI was treated as a participant-level outcome. For eye-specific outcomes, quantitative synthesis required a clearly defined analysis unit, with either one eye included per participant or bilateral-eye data analyzed using methods that appropriately accounted for inter-eye correlation. Measurement procedures also had to be sufficiently comparable across studies for the same outcome. Studies with unclear eye selection or data aggregation, bilateral-eye data without appropriate adjustment for inter-eye correlation, or insufficiently comparable measurement procedures were not included in the corresponding quantitative synthesis. Defined DED classifications were treated as categorical outcomes, whereas MGD and MGA were treated as secondary findings because of differences in outcome definitions across studies.

### 2.5 Methodological quality assessment

Methodological quality was independently assessed by two reviewers using an adapted Newcastle–Ottawa Scale (NOS) based on the original framework (14). Up to nine stars were assigned across selection, comparability, and outcome/exposure ascertainment. Given previously reported concerns regarding inter-reviewer reliability of the NOS (15), stars were awarded only when criteria were explicitly met; nonsignificant baseline differences alone were not considered evidence of adequate comparability. Disagreements were resolved by consensus or third-reviewer adjudication. Item-level ratings and total scores are provided in Supplementary Table S2.

### 2.6 Statistical analyses

Meta-analyses were performed in R version 4.5.2 using the metafor package. Continuous outcomes were summarized as mean differences (MDs) with 95% confidence intervals (CIs), defined as KC minus control. Random-effects models were fitted using restricted maximum likelihood estimation with Hartung-Knapp adjustment. Heterogeneity was assessed using Cochran's Q, I^2^, and *τ*^2^, and a 95% prediction interval was reported for OSDI. Sensitivity and leave-one-out analyses were performed to assess result robustness and the influence of individual studies. Subgroup analyses were conducted only when at least two studies were available per subgroup, with subgroup differences tested using univariable mixed-effects meta-regression with Hartung-Knapp adjustment. Formal tests for funnel-plot asymmetry and trim-and-fill analysis were performed only when sufficient studies were available; otherwise, funnel plots were used for descriptive assessment. All tests were two-sided, with *P* < 0.05 considered statistically significant.

## 3 Results

### 3.1 Study selection

A total of 642 records were identified. After duplicate removal, 405 records underwent title and abstract screening, of which 370 were excluded. Thirty-five articles were assessed in full text, and 28 were excluded. Ultimately, seven studies (16–22) were included in the systematic review and meta-analysis (Figure 1).

**Figure 1:**
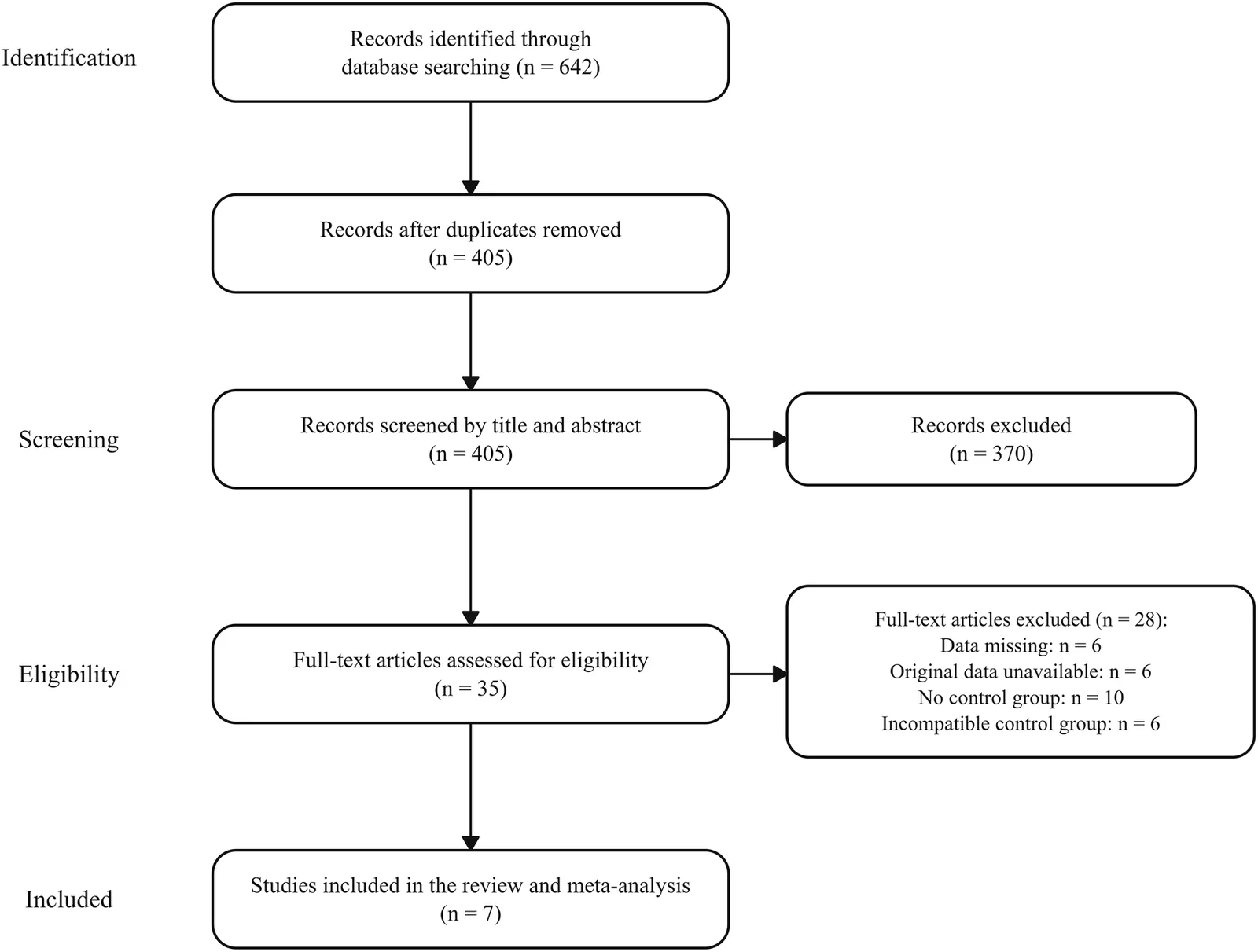
Study selection flowchart.

### 3.2 Study characteristics

Seven studies published between 2019 and 2026 were included, comprising 359 participants with KC and 333 non-KC controls. The studies were conducted in six countries or regions and used cross-sectional, retrospective, prospective, or case-control designs.

Five studies contributed to the OSDI meta-analysis and three to the fluorescein TBUT meta-analysis. Three studies reported NITBUT and five reported SIT; however, quantitative synthesis was not performed for these outcomes because eye-level data handling and/or measurement procedures were insufficiently clear or comparable in several studies. Detailed study characteristics are presented in Table 1, with source-verification details in Supplementary Table S1.

**Table 1:** Characteristics of included studies.

Part A. Study design and participant characteristics								
Study	Country/region	Study design	KC sample	Control sample	Age-KC	Age-Control	Sex-KC	Sex-Control
Constantin et al., 2019 (22)	Romania	Comparative observational study	36 participants; eye count NR	36 participants; eye count NR	26.05 ± 7.88 y (range 13–49)	34.16 ± 10.04 y (range 14–49)	28 M/8 F	18 M/18 F
Mirza et al., 2022 (17)	Türkiye	Prospective comparative study	58 participants/58 study eyes (28 severe; 30 mild)	22 participants/22 study eyes	Severe: 27.8 ± 9.5 y; mild: 26.9 ± 8.5 y	28.0 ± 9.4 y	Severe: 14 F/14 M; mild: 15 F/15 M	11 F/11 M
Uçakhan & Özcan, 2023 (20)	Türkiye	Retrospective comparative study	100 participants/100 eyes	100 participants/100 eyes	22.6 ± 6.0 y (range 11–43)	23.5 ± 6.9 y (range 11–45)	55 M/45 F	55 M/45 F
Morice et al., 2025 (19)	France	Prospective monocentric comparative case-control study	50 participants/88 eyes	46 participants/88 eyes	29 y, median (IQR 23–35)	28 y, median (IQR 26–31)	50% male	50% male
Alshammeri & Alharbi, 2025 (16)	Saudi Arabia	Cross-sectional observational study	17 participants	17 participants	25.9 ± 6.8 y	22.6 ± 8.4 y	NR; groups reported as sex-matched	NR; groups reported as sex-matched
Ifrah & Darwish, 2026 (21)	Palestinian Authority	Retrospective comparative study	17 participants/33 eyes	27 participants/27 right eyes	29.2 ± 7.7 y (range 19–50)	34.2 ± 11.7 y (range 18–56)	8 F (47.1%)	11 F (40.7%)
Narooie-Noori et al., 2026 (18)	Iran	Prospective comparative study	81 participants; final analytic eye count/selection NR	85 participants; final analytic eye count/selection NR	27.54 ± 5.44 y (range 19–38)	27.52 ± 5.63 y (range 20–38)	61.7% male	63.5% male

Part B. Keratoconus definition, ocular-surface eligibility, outcomes, and analysis-unit handling					
Study	KC definition/severity	Contact-lens policy	Control-group definition/ocular-surface selection	Outcomes relevant to this review	Study-eye/analysis-unit handling
Constantin et al., 2019 (22)	Amsler-Krumeich: stage I, *n* = 5; II, *n* = 12; III, *n* = 5; IV, *n* = 14	NR	Controls were patients without keratoconus/ocular disease; no DED-specific selection criterion was stated.	OSDI; fluorescein TBUT; SIT	Participant numbers were reported; the study-eye selection/aggregation for objective tests was not specified.
Mirza et al., 2022 (17)	Rabinowitz-compatible topographic criteria; severe Kmax ≥52.0 D; mild Kmax ≥47.2 and <52.0 D	All participants had never worn contact lenses.	Controls had normal topography and no diagnosed DED or systemic disease.	OSDI; fluorescein TBUT; SIT; conjunctival impression cytology	Higher-Kmax KC eye selected; one randomized control eye selected; one eye/person.
Uçakhan & Özcan, 2023 (20)	Clinical/tomographic KC diagnosis; Amsler-Krumeich system used for severity	Contact-lens use was an exclusion criterion for both groups.	Age-matched controls; both groups excluded history of DED, ocular surgery, rosacea, allergic/atopic/vernal keratoconjunctivitis, or other ocular-surface disease.	OSDI; NITBUT; fluorescein TBUT; SIT; meibography; ocular-surface staining	One eye/person; if both eyes had KC, the more advanced eye was included.
Morice et al., 2025 (19)	Clinical/slit-lamp/topographic diagnosis confirmed by two experts; Amsler-Krumeich severity classification; prior CXL/ocular surgery excluded	Contact-lens wearers were allowed; both groups stopped lenses ≥2 days before examination.	Age- and gender-matched healthy controls; clinical/subclinical KC and other ocular disease excluded. Dermatologic disease/drugs affecting meibum were recorded, not exclusion criteria.	OSDI; SPEED; fluorescein TBUT; meibography/meiboscore (MGA); lipid-layer thickness	Bilateral eyes included; linear/logistic mixed models used random effects for subject and matched set to account for inter-eye correlation and matching.
Alshammeri & Alharbi, 2025 (16)	Pentacam-based grouping: KC Kmax >47.2 D; control Kmax <47.2 D	NR	KC recruited from Qassim University Hospital; controls randomly sampled at Qassim University. Participants with ocular infection, relevant systemic/autoimmune disease, DED treatment, or topical ocular anti-inflammatory use were excluded.	OSDI; NITBUT; tear meniscus height; meibography; lipid-layer evaluation	Grouping was based on the eye with higher Kmax; the paper did not explicitly state whether every ocular-surface endpoint used that same index eye.
Ifrah & Darwish, 2026 (21)	Previously ophthalmologist-diagnosed KC; Amsler-Krumeich: 17 eyes stage 1, 13 stage 2, 0 stage 3, 3 stage 4	Only non-contact-lens wearers were reviewed.	Healthy controls without KC; files with ongoing ocular-surface disease, allergic/atopic/vernal keratoconjunctivitis, relevant systemic medications, or pregnancy were excluded.	OSDI; fluorescein TBUT; SIT; meibography; meibum expressibility/quality; DED; MGD	Both eyes of most KC patients were included (33 eyes from 17 participants); controls contributed right eyes only. Analyses did not model within-participant inter-eye correlation.
Narooie-Noori et al., 2026 (18)	Characteristic topographic/slit-lamp findings; Amsler-Krumeich grades 1–4; 98.77% mild-moderate	Patients who had worn contact lenses within 2 weeks were excluded; controls had no continuous contact-lens wear.	Age-matched healthy relatives with normal corneal topography; no history of ocular surgery or ocular-surface disease.	NITBUT; fluorescein TBUT; SIT	Participant *n* was reported. TBUT testing was repeated three times and averaged for each eye, but the final study-eye selection/aggregation rule was not stated.

KC, keratoconus; DED, dry eye disease; OSDI, Ocular Surface Disease Index; TBUT, tear break-up time; NITBUT, noninvasive tear break-up time; SIT, Schirmer I test; MGA, meibomian gland atrophy; MGD, meibomian gland dysfunction; CXL, corneal cross-linking; NR, not reported.

### 3.3 Methodological quality

Adapted NOS scores ranged from 4 to 7. Alshammeri et al., Morice et al., and Narooie-Noori et al. scored 7; Uçakhan and Özcan scored 6; and Constantin et al., Mirza et al., and Ifrah and Darwish scored 4. Summary scores are presented in Table 2, with item-level assessments and supporting justifications in Supplementary Table S2.

**Table 2:** Methodological quality assessment using an adapted Newcastle-Ottawa scale.

Study	S1	S2	S3	S4	C1	C2	E1	E2	E3	Selection (0–4)	Comparability (0–2)	Outcome (0–3)	Total (0–9)
Constantin et al., 2019 (22)	1	0	0	1	0	0	1	1	0	2	0	2	4
Mirza et al., 2022 (17)	1	0	0	1	0	0	1	1	0	2	0	2	4
Uçakhan & Özcan, 2023 (20)	1	1	0	1	1	0	1	1	0	3	1	2	6
Morice et al., 2025 (19)	1	1	0	1	1	1	1	1	0	3	2	2	7
Alshammeri & Alharbi, 2025 (16)	1	0	1	1	1	1	1	1	0	3	2	2	7
Ifrah & Darwish, 2026 (21)	1	0	0	1	0	0	1	1	0	2	0	2	4
Narooie-Noori et al., 2026 (18)	1	0	1	1	1	1	1	1	0	3	2	2	7

Adapted NOS criteria: S1, adequate KC definition; S2, representativeness of the KC series; S3, appropriate control selection; S4, adequate control definition; C1, age controlled by matching/design/statistical adjustment; C2, sex or another key confounder controlled by matching/statistical adjustment; E1, standardized outcome ascertainment; E2, same ascertainment method in KC and control groups; E3, non-response/completeness adequately demonstrated.

Scoring: 1 = criterion met; 0 = criterion not met. No high/moderate/low quality categories were assigned.

### 3.4 Continuous ocular-surface outcomes

#### 3.4.1 OSDI

Five studies comprising 261 participants with KC and 221 controls contributed participant-level OSDI data. OSDI scores were significantly higher in KC (MD, 19.45 points; 95% CI, 12.27 to 26.63; *P* = 0.0017), with substantial heterogeneity (I^2^ = 79.9%; *τ*^2^ = 25.84). The 95% prediction interval was 3.62 to 35.29 points (Figure 2A).

**Figure 2:**
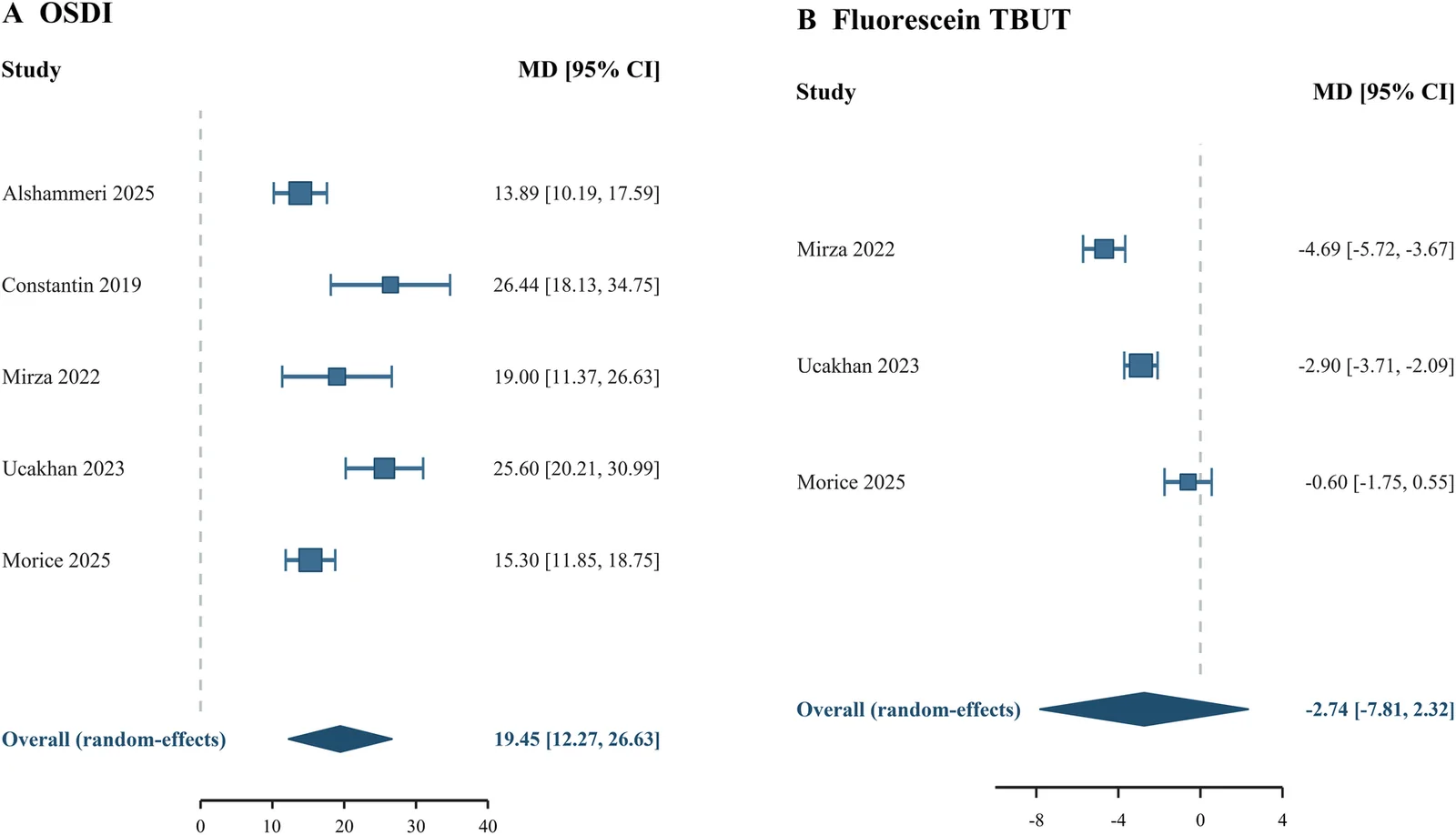
Forest plots of quantitatively pooled ocular-surface outcomes in patients with KC compared with non-KC controls. **(A)** OSDI; **(B)** fluorescein TBUT.

#### 3.4.2 Fluorescein TBUT

Three studies were included in the fluorescein TBUT meta-analysis. Mirza et al. and Uçakhan and Özcan analyzed one eye per participant, whereas Morice et al. included both eyes and accounted for within-participant correlation in the original analysis. Fluorescein TBUT was shorter in KC, although the pooled difference was not statistically significant (MD, −2.74 s; 95% CI, −7.81 to 2.32; *P* = 0.145; I^2^ = 93.8%; *τ*^2^ = 3.85) (Figure 2B).

#### 3.4.3 NITBUT

Three studies reported NITBUT. Uçakhan and Özcan analyzed one eye per participant. In Alshammeri et al., grouping was based on the eye with the higher Kmax, but the study did not clearly state whether the same eye was used for all ocular-surface measurements. Narooie-Noori et al. reported that repeated measurements were averaged for each eye but did not specify the final eye-selection or data-aggregation approach. NITBUT values were lower in the KC group in all three studies. Because the analysis unit could not be clearly established in two of the three studies, a pooled NITBUT estimate was not calculated.

#### 3.4.4 SIT

Five studies reported SIT. Mirza et al. analyzed one eye per participant. Uçakhan and Özcan also analyzed one eye per participant, but the reported Schirmer testing procedure differed from the other studies and was insufficiently specified for direct quantitative comparison. Constantin et al. and Narooie-Noori et al. did not clearly report the final eye-selection or data-aggregation approach, whereas Ifrah and Darwish included 33 KC eyes from 17 participants without adjustment for inter-eye correlation. SIT values were lower in KC in several studies, whereas Uçakhan and Özcan and Ifrah and Darwish reported little or no between-group difference. A pooled SIT estimate was therefore not calculated.

### 3.5 Categorical ocular-surface outcomes

Categorical DED outcomes and secondary MGD/MGA findings were not pooled because they represented distinct and noncomparable categorical endpoints. In Ifrah and Darwish, the prevalence of DED did not differ significantly between KC and control eyes (28/33 vs. 22/27; *P* = 0.74), whereas MGD was significantly more prevalent in KC eyes (22/33 vs. 8/27; *P* = 0.004). Morice et al. reported abnormal MGA (total meiboscore ≥2) in 57/88 KC eyes (64.8%) and 24/88 control eyes (27.3%), with significantly higher odds in KC (adjusted OR, 4.91; 95% CI, 2.56 to 9.38).

### 3.6 Subgroup analysis

OSDI was stratified according to whether control participants were explicitly excluded for DED or ocular-surface disease. The pooled MD was 17.61 points (95% CI, 1.82 to 33.41; k = 3) without explicit exclusion and 22.87 points (95% CI, −18.42 to 64.16; k = 2) with explicit exclusion. No statistically significant difference was observed between subgroups (*P* = 0.403), with substantial residual heterogeneity (I^2^ = 77.0%; *τ*^2^ = 21.56) (Figure 3).

**Figure 3:**
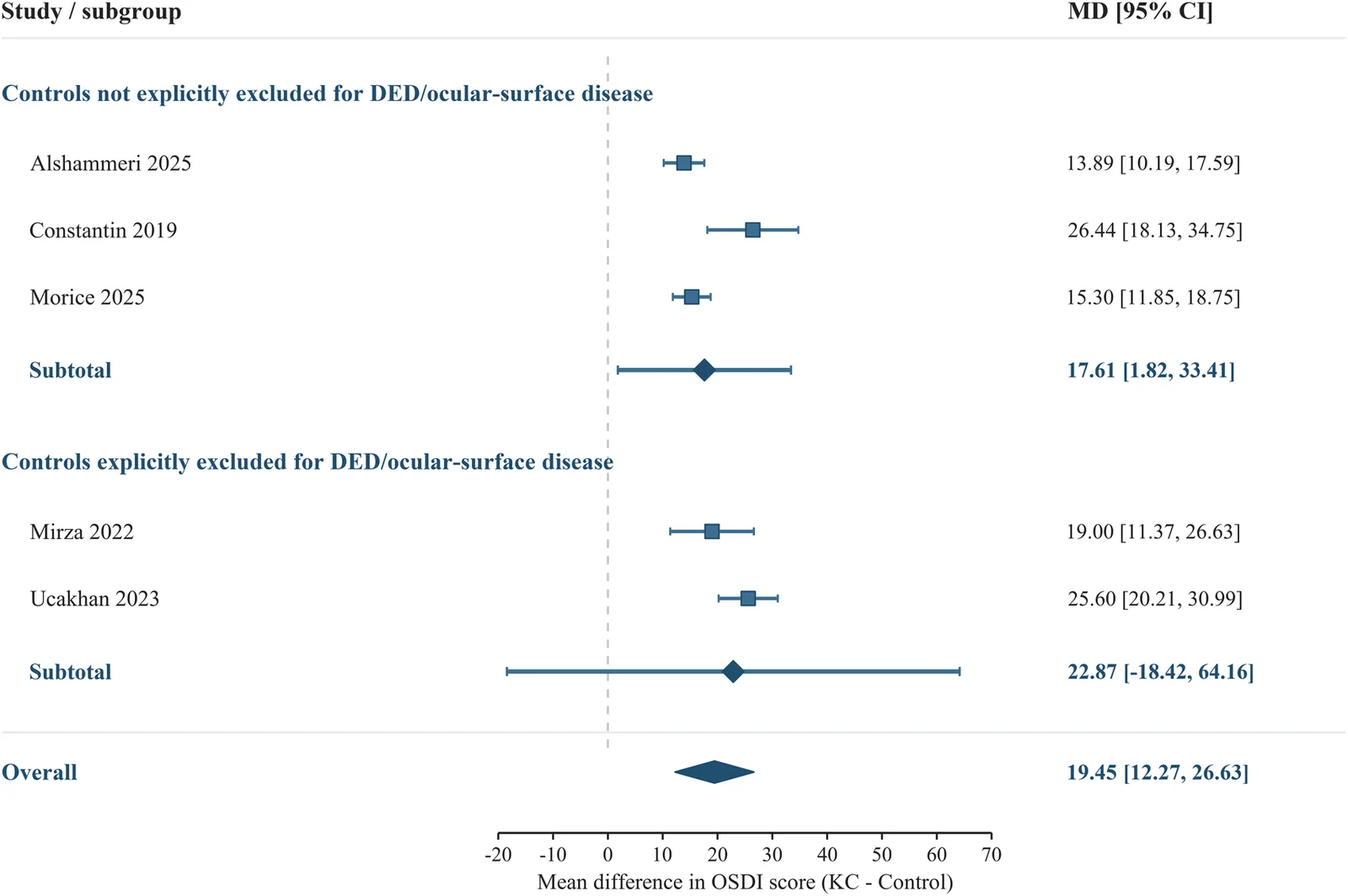
Subgroup analysis of OSDI according to explicit exclusion of dry eye disease or ocular-surface disease in control participants.

### 3.7 Sensitivity and leave-one-out analyses

OSDI remained statistically significant across sensitivity analyses, including exclusion of Constantin et al. or Alshammeri et al. and substitution of the raw participant-level estimate from Morice et al. Leave-one-out analysis showed that all OSDI estimates remained statistically significant (MD range, 17.6 to 21.1 points). For fluorescein TBUT, effect directions remained unchanged in leave-one-out analyses, although statistical significance was not consistently retained after omission of individual studies (Figure 4).

**Figure 4:**
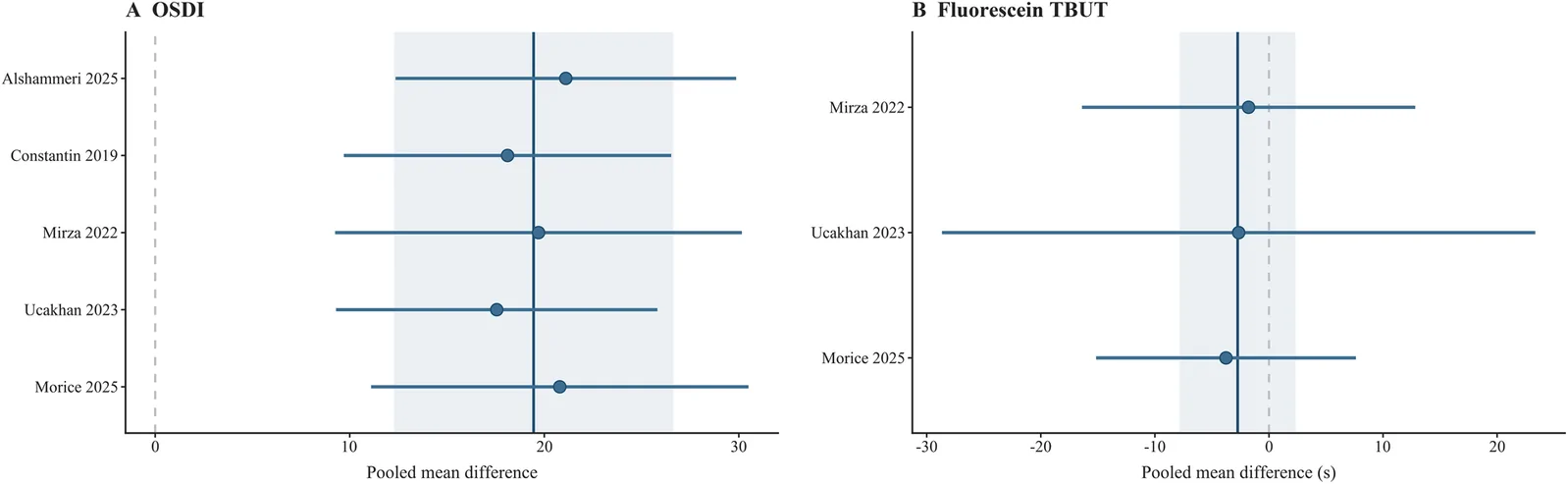
Leave-one-out sensitivity analyses of the quantitatively pooled ocular-surface outcomes. **(A)** OSDI; **(B)** fluorescein TBUT.

### 3.8 Assessment of small-study effects

The OSDI and fluorescein TBUT meta-analyses included only five and three studies, respectively; therefore, formal tests of funnel-plot asymmetry and trim-and-fill analysis were not performed. Funnel plots were presented for descriptive assessment only (Figure 5).

**Figure 5:**
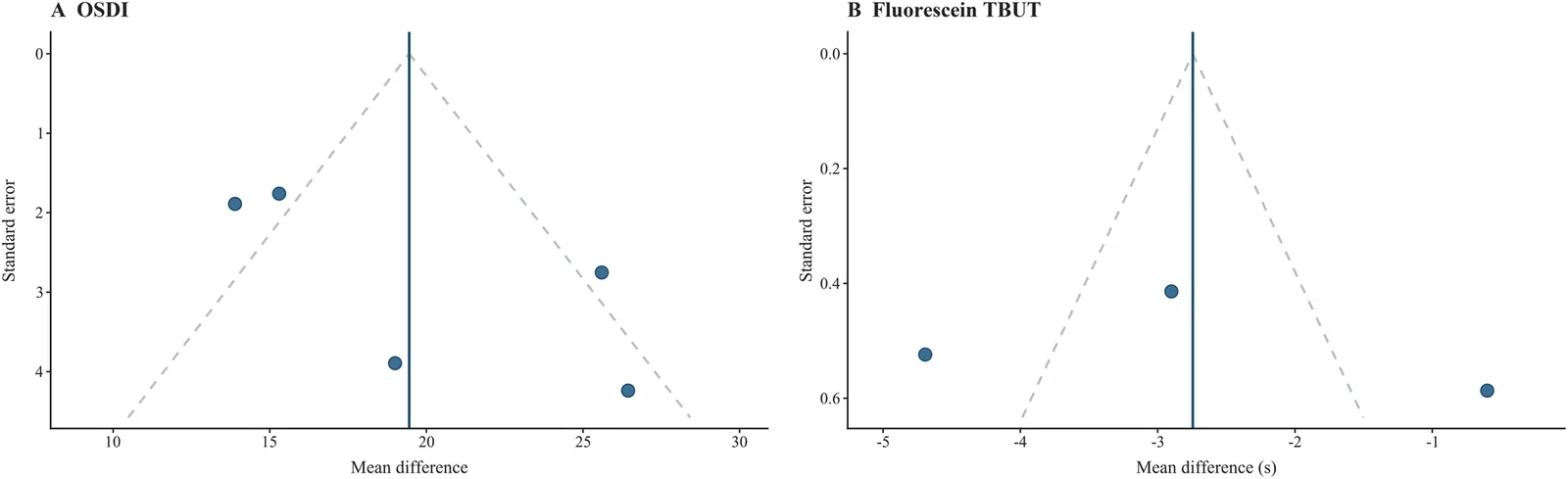
Funnel plots of the quantitatively pooled ocular-surface outcomes. **(A)** OSDI; **(B)** fluorescein TBUT.

## 4 Discussion

This meta-analysis showed a greater ocular-surface symptom burden in patients with KC. OSDI scores were significantly higher in KC and remained robust across sensitivity and leave-one-out analyses. However, OSDI is not specific to DED and may also capture visual fluctuation, irregular corneal optics, contact-lens intolerance, and ocular irritation. No statistically significant subgroup difference was detected according to whether controls with DED or ocular-surface disease were explicitly excluded, although the small number of studies limits interpretation of this comparison. Fluorescein TBUT was shorter in KC, but the pooled difference was not statistically significant. All three studies reporting NITBUT found lower values in KC; however, only one clearly used an independent one-eye-per-participant analysis. SIT findings varied across studies, and differences in eye-level data handling and Schirmer testing procedures prevented a reliable pooled comparison. Thus, the most consistent quantitative finding was the higher OSDI score in KC, whereas tear-film measures showed greater methodological and between-study variability.

Several mechanisms may contribute to the observed ocular-surface findings. Repetitive eye rubbing may promote epithelial injury and extracellular-matrix remodeling (3, 4, 23). In parallel, KC is increasingly recognized as involving an altered inflammatory and oxidative microenvironment rather than being a purely noninflammatory ectasia. Elevated tear concentrations of proinflammatory mediators, including IL-1β, IL-6, TNF-α, and matrix metalloproteinases, have been reported and may promote epithelial dysfunction and stromal matrix degradation (10, 24). Tear proteomic alterations further support disruption of inflammatory, extracellular-matrix, and epithelial homeostatic pathways (25, 26). Ocular-surface structural changes may also contribute: conjunctival cytologic abnormalities and impaired tear function have been observed in KC (27). Meibomian gland dysfunction and gland atrophy have also been reported in KC, although their functional relationship with tear-film stability remains uncertain (19, 28). Together, these observations provide plausible biological mechanisms through which KC may be associated with ocular-surface symptoms and alterations in tear-film-related measures. Nevertheless, the available evidence is predominantly observational and cannot establish whether these changes are causes, consequences, or parallel manifestations of KC.

The categorical findings further emphasize the heterogeneity of ocular-surface involvement in KC. DED classifications and secondary MGD/MGA findings differed substantially in definition and could not be pooled. Future studies should assess symptom burden, tear-film stability, tear secretion, and meibomian gland abnormalities using standardized diagnostic criteria, clearly defined analysis units, and consistent testing protocols.

Several limitations should be acknowledged. Only seven studies were included, with few studies available for each outcome, resulting in limited statistical power and restricting subgroup and small-study-effect analyses. Clinical and methodological heterogeneity was evident in KC severity, contact-lens exposure, allergy or atopy, previous ocular procedures, control selection, and testing protocols. Analysis units were also inconsistently reported. Some studies used one eye per participant or appropriately modeled bilateral-eye data, whereas others did not clearly specify eye selection or data aggregation. This uncertainty limited quantitative synthesis of NITBUT and SIT. In addition, DED definitions and tear-testing protocols were not standardized. Finally, most included studies were single-center observational studies, limiting causal inference and generalizability.

## 5 Conclusion

Current evidence indicates that patients with KC have higher OSDI scores. The pooled difference in fluorescein TBUT was not statistically significant. Lower NITBUT and SIT values were reported in several studies, but pooled estimates for these outcomes were not calculated because of limitations in eye-level data handling and measurement comparability. Larger prospective multicenter studies using standardized DED definitions, clearly specified analysis units, and harmonized tear-testing protocols are needed.

## Data Availability

The original contributions presented in the study are included in the article/Supplementary Material, further inquiries can be directed to the corresponding author.
