# Supplementary material for: Ocular surface symptoms and tear-film parameters in keratoconus: a systematic review and meta-analysis
Source: Front Med (Lausanne). 2026 Oct 15;13:1797492. doi: 10.3389/fmed.2026.1797492 (PMC13716560; doi:10.3389/fmed.2026.1797492)
Supplement: Supplementary file 1 [file Datasheet1.pdf]

Supplementary Table S1. Source verification details for study characteristics

Study	Field checked	Source-verified information	Original article page
Constantin et al., 2019	Age/sex	KC 26.05±7.88 y, 28M/8F; control 34.16±10.04 y, 18M/18F.	p.2
Constantin et al., 2019	Design/sample	72 clinic patients: 36 KC and 36 controls; KC staged with Krumeich–Amsler.	pp.1–2
Constantin et al., 2019	Objective tests	OSDI, Schirmer I test and fluorescein BUT determined for all patients; eye-selection rule not stated.	p.2
Mirza et al., 2022	Age/sex	Severe KC 27.8±9.5 y, 14F/14M; mild KC 26.9±8.5 y, 15F/15M; controls 28±9.4 y, 11F/11M.	p.3
Mirza et al., 2022	Sample/eye rule	58 KC and 22 controls; higher-Kmax KC eye and one randomized control eye were included.	p.2
Mirza et al., 2022	Eligibility	All participants had never worn contact lenses; controls with diagnosed DED/systemic disease were excluded.	p.2
Uçakhan & Özcan, 2023	Age/sex	KC 22.6±6.0 y, 55M/45F; controls 23.5±6.9 y, 55M/45F.	p.3
Uçakhan & Özcan, 2023	Sampling/eye rule	Consecutive KC records reviewed; one eye/person, using the more advanced KC eye if bilateral.	p.2
Uçakhan & Özcan, 2023	Eligibility	History of DED/ocular surgery, contact-lens use, rosacea/allergy/atopy/vernal disease, or other ocular-surface disease excluded in both groups.	p.2
Morice et al., 2025	Age/sex	KC 29 y, median (IQR 23–35), control 28 y, median (IQR 26–31); male proportion 50% in both groups.	p.3
Morice et al., 2025	Sample/eye rule	88 KC eyes from 50 patients and 88 control eyes from 46 patients; bilateral-eye correlation handled with mixed models.	pp.2–3
Morice et al., 2025	Contact lenses/control	Lens wear allowed with ≥2-day washout; controls matched for age/gender and screened for clinical/subclinical KC/other ocular disease.	p.2
Alshammeri & Alharbi, 2025	Age/sex	KC 25.9±6.8 y; control 22.6±8.4 y. Sex counts were not reported; groups were stated to be sex-matched.	pp.2–3
Alshammeri & Alharbi, 2025	Sample/selection	17 KC and 17 controls; KC from QU Hospital, controls randomly sampled at Qassim University; age/sex matched.	p.2
Alshammeri & Alharbi, 2025	Eye rule	Grouping used the higher-Kmax eye; the paper did not explicitly state whether all ocular-surface endpoints used that same eye.	p.2
Ifrah & Darwish, 2026	Age/sex	KC 29.2±7.7 y, 8F/17 (47.1%); control 34.2±11.7 y, 11F/27 (40.7%).	p.4

Study	Field checked	Source-verified information	Original article page
Ifrah & Darwish, 2026	Sample/eye rule	17 KC patients contributed 33 eyes; 27 controls contributed 27 right eyes; no cluster-aware model was described.	pp.3–4
Ifrah & Darwish, 2026	Eligibility	Only non-contact-lens wearers; files with ongoing ocular-surface disease, allergy/atopy/vernal disease, relevant systemic medication use or pregnancy excluded.	p.2
Narooie-Noori et al., 2026	Age/sex	KC 27.54±5.44 y, 61.7% male; controls 27.52±5.63 y, 63.5% male.	pp.1,3
Narooie-Noori et al., 2026	Control/eligibility	Age-matched healthy relatives with normal topography; surgery/ocular-surface disease exclusions; recent contact-lens wear excluded.	p.2
Narooie-Noori et al., 2026	Eye rule	TBUT measurements were averaged for each eye, but the final eye-selection/aggregation rule was not stated.	p.3

Note: KC, keratoconus; DED, dry eye disease; OSDI, Ocular Surface Disease Index; TBUT, tear break-up time; SIT, Schirmer I test; Kmax, maximum keratometry; NR, not reported.
