# Supplementary material for: Ocular surface symptoms and tear-film parameters in keratoconus: a systematic review and meta-analysis
Source: Front Med (Lausanne). 2026 Oct 15;13:1797492. doi: 10.3389/fmed.2026.1797492 (PMC13716560; doi:10.3389/fmed.2026.1797492)
Supplement: Supplementary file 2 [file Datasheet2.pdf]

## Supplementary Table S2. Item-level adapted Newcastle-Ottawa Scale assessment and justification

| Study | NOS item | Star (1/0) | Rationale based on original article | Original article page |
|---|---|---|---|---|
| Constantin et al., 2019 | S1 | 1 | KC stages established using Krumeich–Amsler after complete ophthalmologic examination. | p.2 |
| Constantin et al., 2019 | S2 | 0 | Clinic recruitment reported; consecutive/random KC sampling not reported. | p.2 |
| Constantin et al., 2019 | S3 | 0 | Controls came from the same clinic recruitment source; community/random control sampling not reported. | p.2 |
| Constantin et al., 2019 | S4 | 1 | Control group consisted of patients without keratoconus/ocular disease. | pp.1–2 |
| Constantin et al., 2019 | C1 | 0 | Material age imbalance with no matching/adjustment. | p.2 |
| Constantin et al., 2019 | C2 | 0 | Material sex imbalance with no matching/adjustment. | p.2 |
| Constantin et al., 2019 | E1 | 1 | OSDI, Schirmer I and fluorescein BUT measured using stated procedures. | p.2 |
| Constantin et al., 2019 | E2 | 1 | Same clinical assessments were applied across both groups. | p.2 |
| Constantin et al., 2019 | E3 | 0 | Non-response/completeness not adequately reported. | Full text |
| Mirza et al., 2022 | S1 | 1 | KC diagnosis used Rabinowitz-compatible topographic criteria and Kmax severity groups. | p.2 |
| Mirza et al., 2022 | S2 | 0 | Prospective recruitment stated; consecutive/random KC sampling not stated. | p.2 |
| Mirza et al., 2022 | S3 | 0 | Controls recruited from same ophthalmology department; community/random source not stated. | p.2 |
| Mirza et al., 2022 | S4 | 1 | Controls had normal topography and excluded diagnosed DED/systemic disease. | p.2 |
| Mirza et al., 2022 | C1 | 0 | Age similarity alone was not treated as matching/adjustment. | p.3 |
| Mirza et al., 2022 | C2 | 0 | Sex similarity alone was not treated as matching/adjustment. | p.3 |
| Mirza et al., 2022 | E1 | 1 | Standardized TBUT, Schirmer I, CIC and OSDI assessment. | pp.2–3 |
| Mirza et al., 2022 | E2 | 1 | Same measurement framework applied to KC and controls. | pp.2–3 |

| Study | NOS item | Star (1/0) | Rationale based on original article | Original article page |
|---|---|---|---|---|
| Mirza et al., 2022 | E3 | 0 | Non-response/completeness not adequately reported. | Full text |
| Uçakhan & Özcan, 2023 | S1 | 1 | Biomicroscopic/tomographic diagnosis; Amsler–Krumeich severity grading. | p.2 |
| Uçakhan & Özcan, 2023 | S2 | 1 | Consecutive KC medical records from 2016–2020 were reviewed. | p.2 |
| Uçakhan & Özcan, 2023 | S3 | 0 | Controls were clinic attendees applying for laser correction/contact-lens fitting. | p.2 |
| Uçakhan & Özcan, 2023 | S4 | 1 | Explicit DED/ocular-surface exclusion criteria applied to both groups. | p.2 |
| Uçakhan & Özcan, 2023 | C1 | 1 | Controls explicitly age-matched. | pp.1–2 |
| Uçakhan & Özcan, 2023 | C2 | 0 | Sex matching/adjustment not explicitly stated. | p.3 |
| Uçakhan & Özcan, 2023 | E1 | 1 | Standardized OSDI, NIBUT, meibography, fluorescein TBUT, staining and Schirmer I. | pp.2–3 |
| Uçakhan & Özcan, 2023 | E2 | 1 | Same ocular-surface measurements used in KC and controls. | pp.1–3 |
| Uçakhan & Özcan, 2023 | E3 | 0 | Non-response/completeness not sufficiently reported. | Full text |
| Morice et al., 2025 | S1 | 1 | KC diagnosis confirmed by two experts using clinical/topographic Rabinowitz criteria. | p.2 |
| Morice et al., 2025 | S2 | 1 | KC eyes prospectively and consecutively included at national reference center. | p.2 |
| Morice et al., 2025 | S3 | 0 | Healthy controls were matched but source was not clearly community/random sampling. | p.2 |
| Morice et al., 2025 | S4 | 1 | Controls screened to exclude clinical/subclinical KC and other ocular disease. | p.2 |
| Morice et al., 2025 | C1 | 1 | Age matched ±5 years. | p.2 |
| Morice et al., 2025 | C2 | 1 | Gender matched. | p.2 |
| Morice et al., 2025 | E1 | 1 | Standardized meibography, interferometry, T-BUT and dry-eye questionnaires. | pp.1–3 |
| Morice et al., 2025 | E2 | 1 | Same methods used in cases/controls; matched-set models reported. | p.3 |
| Morice et al., 2025 | E3 | 0 | Eligibility exclusions were reported, but non-response/completeness was not NOS-compatible. | pp.2–3 |
| Alshammeri & Alharbi, 2025 | S1 | 1 | KC/control defined by Pentacam Kmax thresholds with confirmed KC diagnosis. | pp.1–2 |

| Study | NOS item | Star (1/0) | Rationale based on original article | Original article page |
|---|---|---|---|---|
| Alshammeri & Alharbi, 2025 | S2 | 0 | Paper calls sample representative, but consecutive/random KC sampling was not described. | p.2 |
| Alshammeri & Alharbi, 2025 | S3 | 1 | Controls randomly sampled at Qassim University. | p.2 |
| Alshammeri & Alharbi, 2025 | S4 | 1 | Controls defined by Kmax <47.2 D plus study exclusions. | p.2 |
| Alshammeri & Alharbi, 2025 | C1 | 1 | Participants explicitly age-matched. | p.2 |
| Alshammeri & Alharbi, 2025 | C2 | 1 | Participants explicitly sex-matched. | p.2 |
| Alshammeri & Alharbi, 2025 | E1 | 1 | Validated OSDI and calibrated Pentacam/Keratograph under standardized conditions. | pp.2–3 |
| Alshammeri & Alharbi, 2025 | E2 | 1 | Same measurement framework used for both groups. | pp.2–3 |
| Alshammeri & Alharbi, 2025 | E3 | 0 | Withdrawal rights stated, but actual non-response/completeness not adequately reported. | p.3 |
| Ifrah & Darwish, 2026 | S1 | 1 | KC previously diagnosed by ophthalmologist; Amsler–Krumeich severity grading. | pp.2–4 |
| Ifrah & Darwish, 2026 | S2 | 0 | Single-clinic retrospective files; consecutive/random KC sampling not stated. | p.2 |
| Ifrah & Darwish, 2026 | S3 | 0 | Controls were healthy patients from same optometry clinic. | p.2 |
| Ifrah & Darwish, 2026 | S4 | 1 | Healthy controls without KC plus explicit ocular-surface/allergy/medication exclusions. | p.2 |
| Ifrah & Darwish, 2026 | C1 | 0 | No age matching/adjustment; nonsignificant baseline P was not treated as confounder control. | p.4 |
| Ifrah & Darwish, 2026 | C2 | 0 | No sex matching/adjustment reported. | p.4 |
| Ifrah & Darwish, 2026 | E1 | 1 | Defined OSDI, TBUT, meibography, MES/MQS and Schirmer procedures/records. | pp.2–3 |
| Ifrah & Darwish, 2026 | E2 | 1 | Same clinic testing framework used for both groups. | pp.2–3 |
| Ifrah & Darwish, 2026 | E3 | 0 | Record completeness/non-response not adequately demonstrated. | Full text |
| Narooie-Noori et al., 2026 | S1 | 1 | Characteristic topographic/slit-lamp diagnosis; Amsler–Krumeich grades 1–4. | p.2 |
| Narooie-Noori et al., 2026 | S2 | 0 | Prospective recruitment reported; consecutive/random KC sampling not reported. | pp.1–2 |

| Study | NOS item | Star (1/0) | Rationale based on original article | Original article page |
|---|---|---|---|---|
| Narooie-Noori et al., 2026 | S3 | 1 | Age-matched healthy relatives with normal topography served as controls. | pp.1–2 |
| Narooie-Noori et al., 2026 | S4 | 1 | Controls had normal topography and no ocular surgery/ocular-surface disease. | p.2 |
| Narooie-Noori et al., 2026 | C1 | 1 | Age matched; regression considered age as confounder. | pp.1–3 |
| Narooie-Noori et al., 2026 | C2 | 1 | Regression considered sex as confounder. | p.3 |
| Narooie-Noori et al., 2026 | E1 | 1 | Specified standardized NITBUT, fluorescein TBUT and Schirmer procedures. | pp.2–3 |
| Narooie-Noori et al., 2026 | E2 | 1 | Same examination methodology and sequence used across groups. | pp.2–3 |
| Narooie-Noori et al., 2026 | E3 | 0 | Participant flow/non-response insufficiently reported for a star. | Full text |

NOS items: S1, adequate KC definition; S2, representativeness of the KC series; S3, appropriate control selection; S4, adequate control definition; C1, age controlled by matching, design, or statistical adjustment; C2, sex or another key confounder controlled by matching or statistical adjustment; E1, standardized outcome ascertainment; E2, the same ascertainment method used in KC and control groups; E3, non-response/completeness adequately demonstrated. Star: 1 = criterion met; 0 = criterion not met.
